# The Impact of Interferon Lambda 3 Gene Polymorphism on Natural Course and Treatment of Hepatitis C

**DOI:** 10.1155/2012/849373

**Published:** 2012-08-27

**Authors:** F. Bellanti, G. Vendemiale, E. Altomare, G. Serviddio

**Affiliations:** ^1^Department of Medical and Occupational Sciences, C.U.R.E. Centre for Liver Disease Research and Treatment, University of Foggia, Italy; ^2^IRCCS Casa Sollievo della Sofferenza, San Giovanni Rotondo, Foggia, Italy

## Abstract

Host genetic factors may predict the outcome and treatment response in hepatitis C virus (HCV) infection. Very recently, three landmark genome-wide association studies identified single nucleotide polymorphisms near the interleukin 28B (IL28B) region which were more frequent in responders to treatment. IL28B encodes interferon (IFN)**λ**3, a type III IFN involved in host antiviral immunity. Favourable variants of the two most widely studied IL28B polymorphisms, rs12979860 and rs8099917, are strong pretreatment predictors of early viral clearance and sustained viral response in patients with genotype 1 HCV infection. Further investigations have implicated IL28B in the development of chronic HCV infection versus spontaneous resolution of acute infection and suggest that IL28B may be a key factor involved in host immunity against HCV. This paper presents an overview about the biological activity and clinical applications of IL28B, summarizing the available data on its impact on HCV infection. Moreover, the potential usefulness of IFN**λ** in the treatment and natural history of this disease is also discussed.

## 1. Introduction

More than 20 years after the discovery of the hepatitisC virus (HCV) in 1989, it is now well established that HCV infection affects all countries, leading to a major global health problem that requires widespread active interventions for its prevention and control [1]. Prevalence data estimate that 130–170 million persons, or 2-3% of the world population are infected with HCV [2]. HCV infection is characterized by two distinct outcomes: in about 30% of cases, innate and adaptive immune responses achieve a permanent control of infection, referred to as spontaneous HCV clearance; however, most frequently, the host immune responses fail and chronic infection is established [3]. Chronic hepatitis C is highly heterogeneous in clinical presentation and outcomes. This heterogeneity may be dependent on virus genotype but is also largely related to host factors that have been clearly proven to affect the severity and rapidity of disease progression [4]. The successful eradication of HCV in chronically infected patients, defined as a sustained virological response (SVR), is associated with a reduced risk of disease progression. Currently, pegylated interferon (PEG-IFN) plus ribavirin (RBV) is considered the standard of care for chronic hepatitis C, but the rate of SVR is around 50% in patients with HCV genotype 1, the most common genotype [5, 6]. Because PEG-IFN/RBV therapy is costly and often accompanied by several adverse effects, pre-treatment predictions of those patients who are unlikely to benefit from this regimen enables ineffective treatment to be avoided. Viral load and viral genotypes and the stage of liver disease strongly predict the response to HCV treatment [7, 8]. Moreover, host genetic differences may influence the response to HCV treatment. Recently, through a genome-wide association study (GWAS) of patients infected by genotype 1 HCV, it has been reported that single nucleotide polymorphisms (SNPs) linked to the cytokine IFN*λ*3 (also known as IL28B) are strongly associated with a response to PEG-IFN/RBV therapy [9–12]. There has subsequently been rapidly increasing data regarding the significance of the IL28B polymorphism not only in response to therapy but also in spontaneous clearance of acute HCV infection. Clinically available tests then made IL28B genotype testing become part of the standard of care. Genetic analysis of the host may thus predict which patients are more likely to respond to treatment, taking into account that IL28B genotype is only one of many factors that can influence response rates to PEG-IFN/RBV therapy in HCV infection and should be interpreted in the context of other clinical factors predicting SVR.

## 2. The IFN***λ*** Family

**Figure 1:**
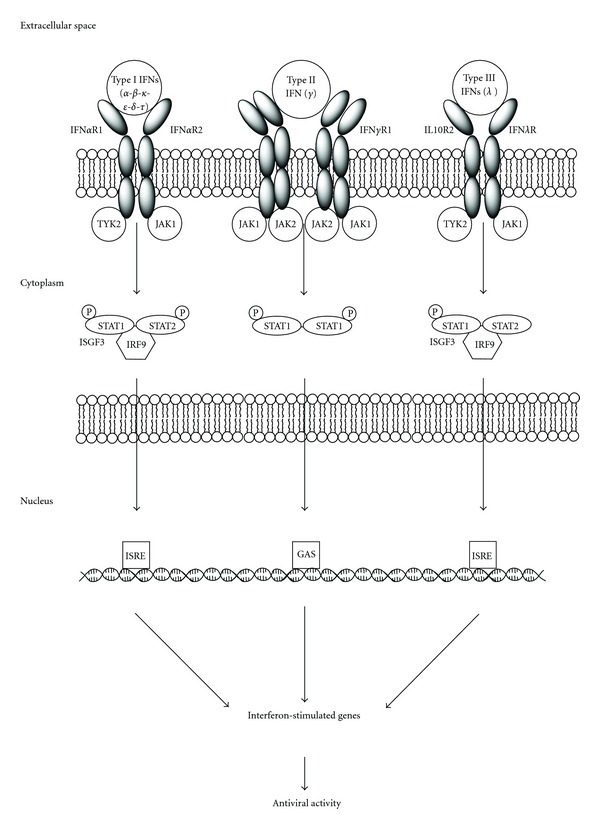
Types I, II, and III IFN receptors and their downstream signalling pathways.

IFN*λ* was identified during the recent years and classified as a new group, type III IFN. The IFN*λ* gene family is composed of three distinct genes: *IFN*λ*1 *(IL29), *IFN*λ*2 *(IL28A), and *IFN*λ*3 *(IL28B) [13, 14]. The members of this IFN family interact through unique receptors that are distinct from type I (IFN**α**/**β**) and type II (IFN**γ**) IFN receptors. The receptor for type III IFN is composed of the unique IFN*λ*R1 chain, also called IL28R*α* and the IL10R2 chain, which is shared with IL10, IL22, and IL26 receptor complexes (Figure 1) [15]. *IL28A*, *IL28B,* and *IL29* are clustered together on chromosome 19 (19q13.13 region) and are coexpressed together with other type I IFNs (IFN*α* and IFN*β*) by virus-infected cells [13, 14]. IFN*λ*Rs are expressed at variable levels on most cell types, and they mediate signalling resulting in induction of many of the same genes that are induced by signalling through IFN*α*/*β* Rs [13]. Very interestingly, hepatocytes from liver biopsy specimens have a high IFN*λ* receptor expression (IL28R*α*), while no expression is evidenced on fibroblasts, endothelial cells, adipocytes, or primary central nervous system cells [16–18].

Signalling through type I (IFN*α*/*β*) or type III (IFN*λ*) IFN receptor complexes results in the formation of a transcription factor complex known as IFN-stimulated gene factor 3 (ISGF3), which consists of three proteins: STAT1, STAT2 and IRF-9 (also known as ISGF3*γ* or p48). After it is fully assembled, ISGF3 translocates to the nucleus where it binds to IFN-stimulated response elements (ISREs) in the promoters of various IFN-stimulated genes classically associated with the antiviral phenotype, including *OAS1*, *MX1*, *IRF7*, and *EIF2AK2* [double-stranded RNA-activated protein kinase (PKR)] [19]. The proteins encoded by these genes mediate the antiviral activity induced by the IFNs [20]. As a result, the downstream biological activities induced by either IFN*α* or IFN*λ* are very similar, including induction of antiviral and antiproliferative activity in many cell types. 

There are currently very limited available data comparing the different biological activity of the three IFN*λ*s. Biological activity ultimately depends on the receptor cytoplasmic domains, which are not related, and these may trigger overlapping but different biological functions. This, in addition to the pattern of receptor distribution among different cell types, means that the different IFN*λ*s are functionally distinct [21]. The effects of IFN*λ*s on immune cell function appear to be complex and diverse, while the antiviral effects are clearer. In fact, IFN*λ*-induced antiviral activity has been demonstrated against many different viruses but has also been shown to inhibit *in vitro* replication of HBV and HCV [22–26]. In particular, it has been demonstrated that IFN*λ*3 inhibits HCV replication in three independent HCV models by the JAK-STAT pathway [27]. The first use of IFN*λ* in clinical setting has started for hepatitis C: pegylated rHu IFN*λ* (PEG-IFN*λ*) has now been evaluated in two phase 1 clinical trials [28, 29]. The initial phase 1A study was designed to evaluate the safety, tolerability, pharmacokinetic, and pharmacodynamic activity of a single dose of PEG-IFN*λ* in healthy volunteers; a few participants developed reversible, dose-related increases in liver transaminases, but PEG-IFN*λ* did not induce fever, fatigue, or any overt haematological changes [28]. The phase 1B study has been conducted in patients with chronic genotype 1 HCV infection, mostly non responders to PEG-IFN/RBV therapy; PEG-IFN*λ* induced significant decreases in the levels of HCV, was well tolerated, and did not induce any significant haematological toxicities such as neutropenia, thrombocytopenia, or anemia [29]. However, this study lacks a direct comparison between IFN*λ* and IFN*α* and the influence of viral and patient genotypes, and it is not clear whether the antiviral effects are mediated directly or through the stimulation of immune cells or both. Since the presumed effects of IFN*λ* on viral clearance observed in the GWAS are mediated through treatment with exogenous IFN*α*, it is conceivable that although IFN*λ* may be less potent than IFN*α* as a direct antiviral, taken together these cytokines may have an additive effect [24].

## 3. The Effect of IFN***λ***3 Polymorphism on IFN***λ*** Biology

IL28B/IFN*λ*3 may harbour the ability to induce potent innate antiviral responses *in vitro* via signalling through the IL28 receptor complex, whose expression has been verified on a variety of cells, including lymphocytes [24, 30, 31]. Moreover, studies performed *in vivo* showed that IL28B/IFN*λ*3 had the potential to induce helper T-cell type 1-biased adaptive cellular immune responses [32]. IFN*λ*3 has significant influence on antigen-specific CD8+ T-cell function, especially in regards to cytotoxicity, since it is a potent effector of the immune system with special emphasis on CD8+ T-cell killing functions [32].

IFN*λ*3 is to date the only member of type III interferons with genetic variations associated with differential expression profiles for downstream genes involved in the immune response and outcome for a disease that exhibits symptoms of dysregulation of the immune response. In 2009, three genome-wide association studies reported an association between SVR and two SNPs located near the gene region encoding IFN*λ*3 (IL28B; rs12980275 and rs8099917) in HCV-infected patients treated with PEG-IFN/RBV combination therapy [9–11]. Further subsequent studies well established that the response to IFN*α* or the natural clearance of HCV infection is dependent on SNPs, upstream of IFN*λ*3, which could be used as biomarkers to help determine the treatment outcome [21]. However, the exact mechanisms by which IFN*λ*3 polymorphisms are identified in the GWAS affect immune function or exert specific antiviral effects in HCV-infected patients are still unclear.

In 2010, two studies performed on HCV-infected patients revealed that both the SNPs of IL28B linked with SVR were strongly associated with lower hepatic expression of interferon-stimulated genes (ISGs) [33, 34]. These results imply that IFN*λ*3 polymorphism may explain the relationship between hepatic ISG expression and HCV treatment outcome but do not reveal any specific mechanism. However, the increase of infected hepatocyte death rate for specific IFN*λ*3 polymorphism suggests that an immune-mediated mechanism may be responsible [35]. 

Further investigations reported some relationships between IL28B genetic variation and allergic disease in children and susceptibility to develop hepatocellular carcinoma in HBV-infected patients [36, 37]. However, even though screening of these polymorphisms and functional studies would be useful to clinical practice for identifying groups at high risk and might help to modify the design of surveillance programs, these studies do not provide any mechanism that explains this association.

In summary, the identity of the functional variants underlying the observed associations is still unknown, and further studies are encouraged to clarify the biological impact of IFN*λ*3 polymorphism.

## 4. The Impact of IFN***λ***3 Polymorphisms on HCV Infection

### 4.1. rs12979860 Polymorphism

The SNP on chromosome 19q13 (rs12979860) strongly associated with SVR in genotype 1 HCV-infected subjects, identified by the study of Ge et al., results in three possible genotypes: the C/C genotype was associated with 2.5 or greater rate (depending on ethnicity) of SVR compared to the T/T genotype, and the C allele was overrepresented in a random multiethnic population as compared to the chronically infected study cohort, raising the possibility that the C allele may favour spontaneous clearance of HCV [11]. According to this report, the rs12979860 polymorphism also may explain much of the difference in response between different population groups: in fact, the genotype leading to better response presents with greater frequency in European than in African populations [11]. Moreover, the C allele was found associated with more subjective appetite, energy, and sleep complaints, as well as lower serum triglycerides and higher serum LDL cholesterol but less hepatic steatosis [38–40]. This latter report was confirmed by a subsequent study, which showed a lower steatosis severity grade in HCV-infected patients with C/C genotype [41]. This association was then observed in HCV genotypes 2, 3, and 4, and also in HCV/HIV-infected patients even though prior non-responders [42–46]. The C allele also appears to affect positively early viral kinetics in patients with chronic hepatitis C receiving interferon-free treatment [47, 48]. However, the C allele is associated with more pronounced liver histopathology damage in patients chronically infected with HCV genotype 3, which may be secondary to higher viral load but has no impact among genotype 2 infected patients, implying that IL28B may differentially regulate the course of genotype 2 and 3 infection [49, 50]. In an analysis of HIV-infected patients with acute hepatitis C, the C/C genotype was associated with higher serum levels of hepatitis C virus RNA, and lower *γ*GT and CD4 cell count, but not significantly associated with treatment response rates, suggesting that its effects would be different in HIV-infected patients with chronic and acute hepatitis C [51]. The C/C genotype predicts SVR in chronically coinfected patients, but it is also associated with higher all-cause mortality [52, 53]. When this variant was genotyped in HCV cohorts comprised of individuals who spontaneously cleared the virus or had persistent infection, the C/C genotype strongly enhanced resolution of HCV infection amongst individuals of both European and African ancestry, showing that IL28B plays a determinant role in natural clearance of HCV and spontaneous resolution of HCV infection [54]. This report was also observed in women affected by acute hepatitis C [55]. However, rs12979860 homozygosity is not associated with resistance to HCV infection in exposed uninfected patients [56]. Studying the impact of donor and recipient genotypes of IL28B rs12979860C>T SNP on hepatitis C virus (HCV) liver graft reinfection revealed a dominant but not exclusive impact of the donor rather than the recipient genetic background on the natural course and treatment outcome [57]. Interestingly, the risk of developing posttransplant diabetes mellitus is significantly increased in recipients carrying the IL28B rs12979860C>T SNP [58].

### 4.2. rs8099917 Polymorphism

The study by Rauch et al. demonstrated that the rs8099917 minor allele (T/G or T/T) was associated with progression to chronic HCV infection and also with failure to respond to therapy, with the strongest effects in patients with HCV genotype 1, 2, or 4 [12, 59]. However, an analysis performed on Taiwanese patients with a lower daily viral production rate than Western patients, demonstrated that the T/T genotype may contribute to the increased viral clearance rate and better virological responses in these patients [60]. Even though there are no studies considering multiethnic cohorts, a recent meta-analysis evidenced that rs8099917 T/T had slight predictive value in Asian patients [61]. A further study reported that combination analyses of SNP of rs8099917 in recipient and donor tissues and mutations in HCV RNA allowed prediction of SVR to PEG-IFN/RBV therapy in patients with recurrent HCV infection after orthotopic liver transplantation [62]. Another study revealed the high prevalence of the rs8099917 G allele in HCV/HIV-1-coinfected patients as well as its strong association with treatment failure in HCV genotype 1-infected patients [63]. The rs8099917 T/T genotype is associated with higher levels of apoB-100 and LDL cholesterol in genotype 1-HCV-infected patients [64].

### 4.3. Combined IFN3*λ* Polymorphisms

Both favourable genotypes for rs12979860 (C/C) and rs8099917 (T/T) were associated with spontaneous HCV clearance, possibly interacting in synergy with female sex [65]. On the other side, IL28B variants associated with poor response to interferon therapy may predict slower fibrosis progression, especially in patients infected with non-1 HCV genotypes [66].

Concerning HCV genotype 3-infected subjects, IL28B polymorphisms are associated with RVR but not SVR to PEG-IFN therapy [67]; other studies showed that IL28B polymorphisms are strongly associated with the first phase viral decline during PEG-IFN/RBV therapy of chronic HCV infection, irrespective of HCV genotype, and in genotype 1–4 HIV/HCV-coinfected patients [66, 68, 69]. These reports suggest that IL28B polymorphisms could play a role in blocking the production or release of virions in the first phase viral decline [70]. Treatment outcome in HCV-infected patients may be also influenced by viral polymorphisms within the viral core and NS5A proteins, even though it has been clearly demonstrated that IL28B polymorphisms and HCV core amino acid 70 substitutions contribute independently to an SVR to PEG-INF/RBV therapy [71]. Another independent predictor of RVR and final therapeutic outcome is IFN*γ* inducible protein-10 (IP-10), and the concomitant assessment of pretreatment IP-10 and IL28B-related SNPs has been proposed to improve response or spontaneous clearance prediction [72–74].

When ten SNPs of *IL28B* were simultaneously analysed in treatment-naïve patients with genotype 1-HCV chronic infection who received PEG-IFN/RBV, rs12979860 resulted as the critical predictor for SVR even in patients without RVR [75]. A further study demonstrated that SNPs rs8099917 and rs12979860 used alone may be useful for predicting the outcome of HCV treatment, and in a rational and cost-effective approach, determination of only one of these two SNPs is sufficient for predicting SVR. Because of the highest predictive SVR associated with rs12979860 C/C compared with the rs8099917 T/T, rs12979860 determination alone is sufficient for predicting interferon response [76, 77], even in HIV/HCV-coinfected patients [78]. However, there is evidence that a significant proportion of heterozygous carriers of the rs12979860 T nonresponder allele can profit with respect to SVR prediction by further determination of the rs8099917 SNPs [79].

When the combined effect of variants of IL28B with human leukocyte antigen C (HLA-C) and its ligands the killer immunoglobulin-like receptors (KIRs), which have previously been implicated in HCV viral control, was studied, prediction of HCV treatment response improved, supporting a role for natural killer (NK) cell activation in PEG-IFN/RBV treatment-induced clearance, partially mediated by IL28B [80]. Nevertheless, there are conflicting reports on the concept that IFN*λ*3 might directly act on NK cells to modify their functional activity [81, 82].

### 4.4. IFN3*λ* Polymorphisms and Directly Acting Antivirals (DAAs)

HCV therapy has been recently updated by the approval of two DAAs against the NS3/4A serine protease for use in genotype 1, the ketoamide inhibitors boceprevir and telaprevir [83, 84]. The impact of IFN3*λ* polymorphisms on therapy outcome has been evaluated in several phase 3 clinical trials of boceprevir and telaprevir when used in conjunction with PEG-IFN/RBV. The predictive factors of SVR to a regimen of PEG-IFN/RBV/telaprevir therapy was evaluated in 72 Japanese adults infected with HCV genotype 1, showing rs8099917 T/T as significant determinant of SVR [85]. In the Phase 3 ADVANCE trial, the addition of telaprevir to PEG-IFN/RBV improved SVR across all rs12979860 genotypes [86]. The retrospective analyses from the SPRINT II and RESPOND-2 trials suggest that patients with the rs12979860 C/C genotype are highly likely to be treated for just 6 months with PEG-IFN/RBV/boceprevir [87].

Several other classes of DAAs are under development, and it is expected that they will increase SVR rates and decrease the required duration of therapy [88]. The role of IL28B variations in predicting response to triple-therapy regimens including DAAs different from boceprevir and telaprevir has very recently been investigated, but it is difficult to draw firm conclusions owing to the small number of patients in some groups, and additional research is required before final conclusions can be drawn [89]. Finally, the rs12979860 C/C genotype presented a favourable influence on early viral kinetics during treatment with an interferon-free DAA regimen (mericitabine plus danoprevir), even though the importance of IL28B genotype on response to future interferon-free combination DAA regimens remains to be determined with analyses of larger and longer duration studies [47] (refer to the Table 1 for a comprehensive summary of the effects).

## 5. Conclusion

The IFN*λ*3 gene polymorphism closely associates with the natural course and treatment response of chronic hepatitis C in different populations, irrespective of HCV genotype, thus it can be considered an important predictive pretreatment factor. The differential global distribution of IFN*λ*3 SNPs may explain the observed clinical differences between ethnic groups. The identification of these polymorphisms will provide support for clinical decision making in current standard care. However, in genotype 2/3 patients who achieve RVR or in treatment initiated in acute HCV, IFN*λ*3 SNPs testing may have less clinical utility. This raises questions about justifying the cost of incorporating IFN*λ*3 SNPs testing in routine practice in such patients. Furthermore, the effect of the IL28B genotype is not absolute, and it should not be used as a criterion for denying therapy when unfavourable.

Future studies are needed to establish the role of IFN*λ*3 genotype using direct antivirals, which rapidly reduce the viral load and may therefore lower the influence of IL28B genotyping in predicting SVR. Further functional studies of IFN*λ*s and the significant SNPs should be investigated to improve the positive predictive value using the point mutation analysis of the targeted polymorphisms. For applying a practical tailor-made therapy, it is also necessary to reveal the cause of exceptional cases that do not follow the IL28B genotyping.

## Figures and Tables

**Table 1:** Summary of the impact of different IL28B polymorphisms on HCV infection in several conditions (SVR: sustained virological response; RVR: rapid virological response).

Polymorphism	Genotype	Impact on HCV	Impact on HCV/HIV	Impact on liver graft reinfection
rs12979860	C/C	(i) Higher SVR (genotypes 1, 2, 3, 4) [11, 41–45] (ii) Spontaneous clearance [11] (iii) Early viral kinetics [46, 47]	(i) Higher SVR [51] (ii) No influence on acute HCV infection [50] (iii) Higher all-cause mortality [52, 53]	(i) Natural course and treatment outcome dependent on donor rather than recipient genetic background [54] (ii) Higher frequency of posttransplant diabetes mellitus [55]

rs8099917	T/G or T/T	(i) Treatment failure (genotypes 1, 2, 4) [12, 56] (ii) Increased viral clearance and virological response in Taiwanese patients [57]		(i) SVR in patients with recurrent HCV infection [58]
	G/G		(i) High prevalence [59] (ii) Treatment failure (genotype 1) [60]	

Combined	C/C and T/T	(i) Spontaneous clearance [61] (ii) Early viral kinetics [62, 64, 65] (iii) RVR but not SVR (genotype 3) [63]	(i) Early viral kinetics (genotypes 1, 4) [65]	
